# Exceptional responders to immunotherapy in pancreatic cancer: A multi-institutional case series of a rare occurrence

**DOI:** 10.18632/oncotarget.28739

**Published:** 2025-06-10

**Authors:** Kavin Sugumar, Andrew Alabd, Andre Alabd, Jonathan J. Hue, Josh Lyons, Sherri Fields, Zev Wainberg, Lei Zheng, Brianna Coogle, Anup Kasi, Nicholas Grewal, Hedy L. Kindler, Jason Starr, Ashwin R. Sama, Jordan M. Winter

**Affiliations:** ^1^Department of Surgery, University Hospitals Seidman Cancer Center, Cleveland, OH 44106, USA; ^2^Department of Medicine, Cooper University Healthcare, Camden, NJ 08103, USA; ^3^Department of Urology, University of Indiana, Indianapolis, IN 46227, USA; ^4^Department of Medicine, UCLA/Santa Monica Cancer Center, CA 90404, USA; ^5^Department of Medicine, Johns Hopkins University, Baltimore, MD 21218, USA; ^6^Department of Medicine, University of Kansas Medical Center, Kansas City, KS 66103, USA; ^7^Department of Medicine, University of Chicago, Chicago, IL 60637, USA; ^8^Department of Medicine, Mayo Clinic, Jacksonville, FL 32224, USA; ^9^Department of Medicine, Jefferson Medical Oncology Associates, Philadelphia, PA 19107, USA

**Keywords:** pancreatic adenocarcinoma, immunotherapy, exceptional responders, microsatellite instability, survival

## Abstract

Introduction: Immunotherapy has emerged as a standard treatment option for multiple solid tumors. However, most patients with pancreatic cancer (PC) do not derive a significant benefit. Identification and analyses of exceptional responders could eventually offer hints as to why PC is resistant to immunotherapy.

Methods: Oncologists from cancer centers in the United States were contacted to identify patients with PC who responded to immunotherapy. Exceptional responders were defined as those having either partial (PR) or complete response (CR) based on Response Evaluation Criteria in Solid Tumors, or biochemical response (CA 19-9 levels) after starting immunotherapy. Patients receiving concurrent chemotherapy were excluded.

Results: 14 patients met inclusion criteria. Immunotherapy drugs included checkpoint inhibitors and macrophage inhibitors. Eight patients (42%) were MSI (microsatellite instability)-high. Radiologically, 82% had PR. Four patients (28%) had marked reduction in CA 19-9. The median progression-free survival was 12 months from the start of immunotherapy. Median survival was not reached. The 1- and 2-year survival probabilities were 80%, 70% respectively.

Conclusion: Majority of clinical trials evaluating immunotherapy in PC have yielded disappointing response rates compared to other solid tumors. Our case series adds to published data from early-phase trials supporting the promise of immunotherapy in some patients with PC.

## INTRODUCTION

Pancreatic cancer (PC) is one of the most aggressive malignancies, with a 5-year survival rate of 13% among all-comers, and just 3% for patients with metastatic disease [1]. It is the third most common cause of cancer-related death in the Unites States, with 50,550 deaths reported in 2023, but is nearing colon cancer with the potential to become the second leading cause [2]. At diagnosis, only 20% and 3% of pancreatic ductal adenocarcinoma (PDAC) in the head and tail of the pancreas are considered candidates for resection, respectively, suggesting that a minority of patients have a substantial probability for long-term survival [3, 4]. The last several decades of research and investments of several billion dollars have failed to move the needle in the treatment paradigm or survival outcomes. Multiagent chemotherapy remains the standard of care for advanced pancreatic cancer [5], and these treatments offer a median survival under one year [6]. While mutation-targeted therapies have improved outcomes for many cancer types, PARP (poly (adenosine diphosphate-ribose) polymerase) inhibition is the only immunotherapy used routinely for PC. However, olaparib yields a modest progression-free survival benefit, without an overall survival benefit, and is indicated for only a minority of patients with BRCA-mutant tumors (<10% of all PC patients) [7].

Advances in immuno-oncology have led to a paradigm shift in the care of many cancer patients. Several immunotherapy drugs including those that block programmed cell death 1 ligand (PD-1), programmed cell death ligand 1(PD-L1), and cytotoxic T lymphocyte-associated protein 4 (CTLA-4) are now indicated as first- or second-line therapy for many solid tumors including melanoma, non-small cell lung cancer, bladder cancer, and renal cell cancer among others [8]. This has led to an increasing interest in evaluating immunotherapy for PC. Indeed, nearly one-third of all active therapy interventional clinical trials in PC are investigating immunotherapeutics (more than 100 in total) [9]. Yet, there has been no evidence that these drugs work against the majority of pancreatic cancers. At present, pembrolizumab, a PD-1 checkpoint inhibitor, is Food and Drug Administration (FDA) approved for treatment of patients with solid tumors that exhibit deficient mismatch repair (dMMR) status and/or high tumor mutational burden (i.e., TMB ≥10 mut/Mb) and/or high microsatellite instability (MSI-H), and who have demonstrated progression of disease with conventional therapy [10, 11]. However, this subset of patient comprises just 1–2% of all PDAC [12].

Despite promising results of immunotherapy in other cancer types, published clinical trials reveal that single agent immunotherapy with checkpoint inhibitors are ineffective against PC [13–15], and outcomes with patients harboring mutations in mismatch repair genes is sparse due to the rarity of the genetic abnormality. Resistance to immunotherapy has been attributed to poor intrinsic antigenicity, defective antigen presentation, an immunosuppressive microenvironment, and suppression of immune cells by PC tumor cells. Nearly 50% of the tumor microenvironment (TME) is comprised of immune cells, but the TME is enriched with myeloid-derived suppressor (MDSC) and regulatory T cells (Treg), which create immune tolerance or escape [16, 17]. To improve patient selection for immunotherapy, several biomarkers have been investigated to predict response to therapy in PC, however none have proven sufficiently effective to warrant routine clinical use [18].

Few case reports of exceptional responders exist [19–21] and most trials to date have combined immunotherapeutics with conventional chemotherapy, which confound the interpretation of treatment response. In this report we take assemble data from patients across institutions to organize the collective experience of exceptional responders to immunotherapy. Despite its small size, this cohort is larger than previously reported studies, which can provide valuable insights into patterns of pertinent variables and long-term outcomes. This can yield clues into what separates this group apart from other patients with PC [18].

## RESULTS

### Patient characteristics

**Figure 1:**
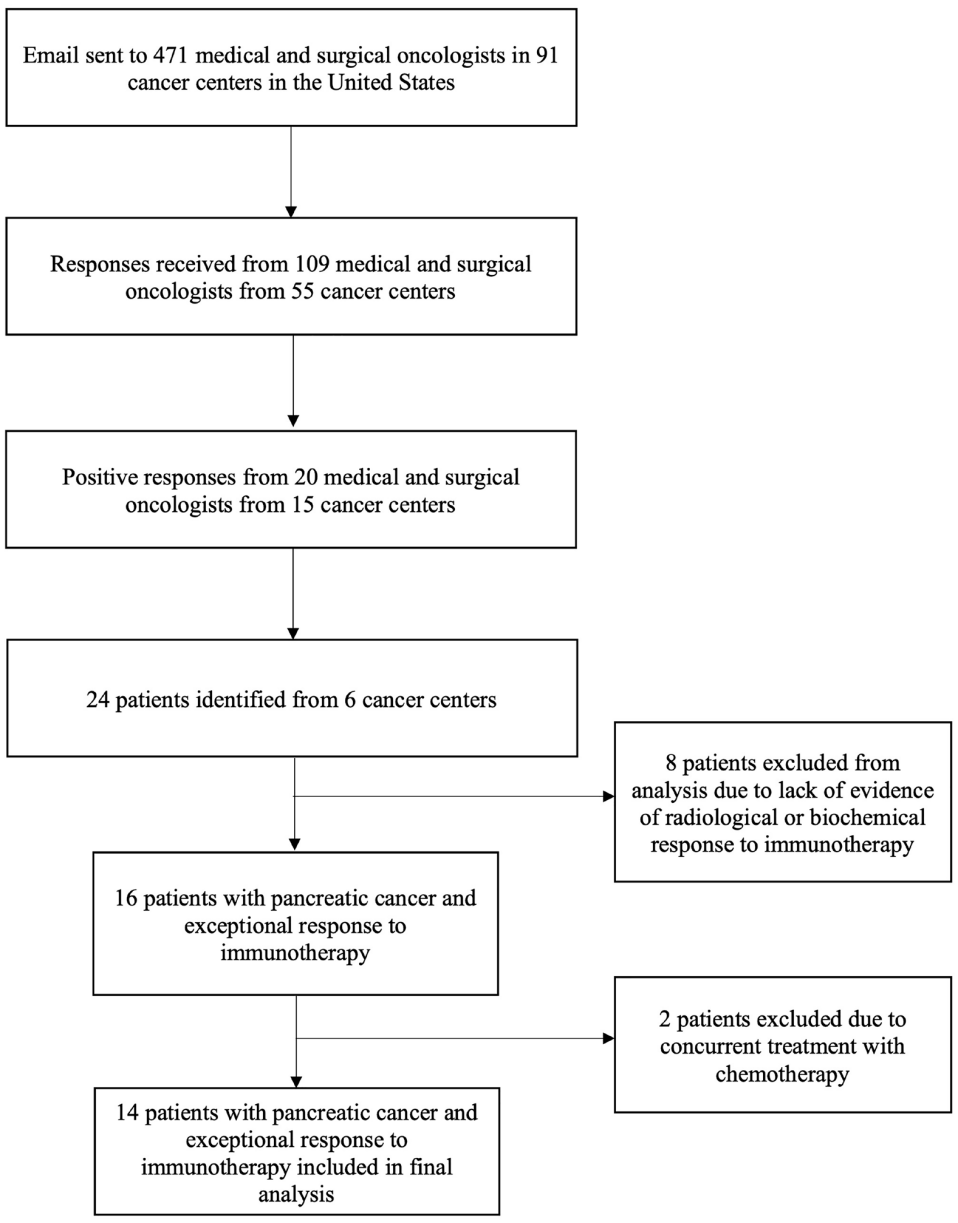
Study flow diagram.

Between 2020–21, 471 oncologists from 91 major cancer centers in the United States were contacted (Figure 1). 109 oncologists responded from 55 cancer centers, of which 20 oncologists reported having treated patients who showed an exceptional response. A total of 24 patients were identified from 6 centers, of which 8 patients were excluded due to lack of verified radiological or biochemical response to immunotherapy on further review. Two patients were excluded due to concurrent therapy with chemotherapy. The final cohort was comprised of 14 patients.

Baseline patient characteristics are shown in Table 1. The most common pathological diagnosis was PDAC (64%, 9/14 patients), followed by intraductal tubular neoplasm with invasive carcinoma (14%, 2/14 patients), intraductal papillary mucinous neoplasm (IPMN) with invasive carcinoma (7%, 1/14 patients), acinar cell cystadenocarcinoma (7%, 1/14 patients), and mucinous cystic neoplasm with invasive carcinoma (7%, 1/14 patients). 42% of patients were MSI-high, while the remainder had MSI-stable disease. BRCA (a marker of homologous recombination repair and with some correlation with immune response [22]) status was not known. All patients had a good functional status at diagnosis with ECOG scores of 0–1.

**Table 1:** Patient characteristics

Patient identifier	1	2	3	4	5	6	7	8	9	10	11	12	13	14
Institution	JHU	JHU	JHU	Jeff	UCLA	UCLA	UCLA	UCLA	UCLA	UCLA	KU	KU	Mayo	UChicago
Age (years)	72	66	53	66	77	63	62	67	69	71	51	70	77	60
Gender	Male	Female	Female	Male	Female	Male	Male	Female	Female	Male	Male	Female	Female	Female
Race	White	White	Black	Hispanic	White	White	White	White	Asian	White	White	White	White	Native Hawaiian
Tissue diagnosis	PDAC	PDAC	PDAC	PDAC	PDAC	PAC	ITN-C	IPMN-C	MCN-C	PDAC	PDAC	ITN-C	PDAC	PDAC
Genetic mutation					MSH2	MSH2, MSH6	MSH2, MSH6		CDX2, SMAD4		KRAS, RNF43	TP53, NF1, CDK12, E102K (MLH1)		
MSI status	High	High	Stable	Stable	High	High	High	Stable	High	Stable	Stable	Stable	Stable	Stable
Diabetes	II	No	No	II	No	No	II	No	No	No	I	No	II	No
Previous cancer	Colon	Colon	No	No	Other	No	No	No	No	No	No	No	No	No
Family history of cancer	Ovarian	Melanoma	Other	No	No	Lung	Colon	Renal	No	Pancreas, Breast	Lung	NHL	No	No
Smoking history	Past	Past	No	No	No	No	No	No	No	No	Current	No	No	No
Stage at diagnosis	IV	III	IV	III	III	III	II	I	I	II	IV	IV	II	IV
ECOG	1	1	0	1	0	1	0	1	1	1	0	0	1	1
Neoadjuvant therapy	No	Yes	No	No	Yes	Yes	No	Yes	Yes	No	No	No	No	No
Chemotherapy (number of cycles)		Gem, Pac (8)			FOLFIRI-NOX (5)	Gem, Pac, 5-FU, Ox		Gem, Cap	FOLFIRI-NOX (9)					
Radiation	No	Yes (3300)	No	No	No	No	No	No	No	No	No	No	No	No
Surgery	No	No	No	Yes	No	No	Yes	Yes	Yes	Yes	No	No	Yes	No
Margin				R1			R0	R0	Unk	Unk			R0	
Lymph node status				10/13 (77%)			Unk	0/31 (0%)	Unk	2/13 (15%)			2/20 (10%)	
Grade	G2	G2	G3	G2	G2	G1	G3	G1	G1	G2	G3		G1	G1
Venous/lymphatic invasion				Yes			Yes	No	No	Yes			No	
Perineural invasion				Yes			No	No	No	No			Yes	
Adjuvant therapy	No	No	No	Yes	No	No	Yes	Yes	Yes	Yes	No	No	Yes	No
Chemotherapy (number or cycles)				FOLFIRI-NOX (24), Cap (1)			FOLFIRI-NOX (7)	Gem, 5-FU, Ox	Gem, Pac (4)	Gem, Cap, FOLFOX (2)			Gem (2)	
Radiation	No	No	No	Yes	No	No	No	No	No	Yes	No	No	No	No
Completion of therapy				Yes			Yes	Yes	Yes	Yes			No	
Palliative therapy	Yes	No	Yes	No	No	No	No	No	No	No	Yes	Yes	No	Yes
Prior 1st line therapy	FOLFIRI-NOX (6)		FOLFOX (8)								FOLFIRI-NOX (9)	FOLFIRI-NOX (7)		FOLFIRI-NOX (12)
2nd line therapy			Cap (6)								FOLFIRI-NOX (6)	Cap (17)		
3rd line therapy												Gem, Pac		
Stage of disease at start of immuno	IV	III	IV	Recurrent disease	III	III	Recurrent disease	Recurrent disease	Recurrent disease	Recurrent disease	IV	IV	Recurrent disease	IV
Immuno	Yes	Yes	Yes	Yes	Yes	Yes	Yes	Yes	Yes	Yes	Yes	Yes	Yes	Yes
Drug used	Ipi, Niv	Pem	Pem	Cab, Niv	Pem	Pem	Pem	Cab	Niv	Cab, Niv	Pem	Pem	Ipi, Niv	Atez
Indication	Prog	Trial	Prog	Trial	Trial	Prog	Trial	Trial	Rec	Rec	Trial	Trial	Trial	Trial
PFS (months)	-	14.3	1.16	14.06	10.63	39	27.5	7	10	14.4	4.3	0.9	36.8	12.26
Progression	-	No	Yes	Yes	Yes	No	Yes	Yes	Yes	Yes	Yes	Yes	No	Yes
OS (months)	12.6	14.3	4	18	11.4	39	27.7	30.03	11.9	15.26	7	3	36.8	16.9
Vital status	Dead	Alive	Alive	Alive	Alive	Alive	Alive	Alive	Dead	Alive	Alive	Alive	Alive	Dead

Abbreviations: JHU: John Hopkins University; Jeff: Thomas Jefferson University; UCLA: University of California, Los Angeles; KU: Kansas University; UChicago: University of Chicago; PDAC: pancreatic ductal adenocarcinoma; PAC: acinar cell cystadenocarcinoma; IPMN-C: intraductal papillary mucinous neoplasm with invasive carcinoma; MCN-C: Mucinous cystic neoplasm with invasive carcinoma; ITN-C: Intraductal tubular neoplasm with invasive carcinoma; MSI: microsatellite instability; ECOG: Eastern Cooperative Oncology Group; NHL: Non-Hodgkin’s lymphoma; FOLFIRINOX: 5-Flourouracil (5-FU), Oxalplatin, Irinotecan; Unk: unknown; Gem: gemcitabine; Cap: capecitabine; Pac: paclitaxel; Ox: Oxaliplatin; Ipi: ipilimumab; Niv: nivolumab; Pem: pembrolizumab; Cab: Cabiralizumab; Atez: Atezolumab; Prog: disease progression; Rec: disease recurrence; Immuno: immunotherapy.

**Figure 2:**
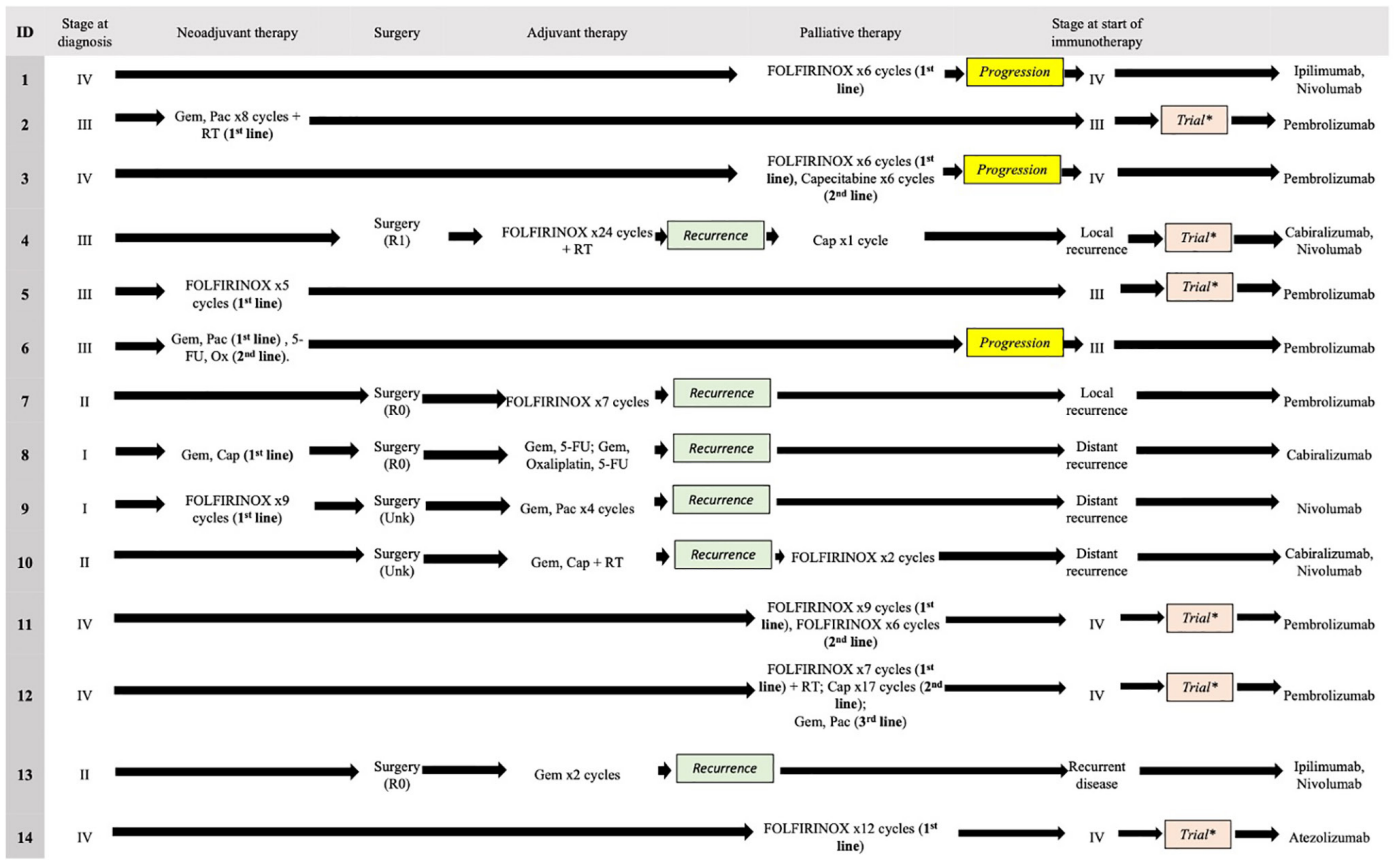
Sequence of treatment. Abbreviations: Unk: Unknown; FOLFIRINOX: 5-Flourouracil (5-FU), Oxaliplatin, Irinotecan, RT: radiotherapy; Cap: capecitabine; Pac: paclitaxel; Ox: Oxaliplatin. ^*^Patient was started on immunotherapy as a part of a trial.

The treatment sequences for all 14 patients are described in Figure 2. Of the 5 patients with stage I–II disease at diagnosis, 60% (3/5 patients) received upfront surgery and 40% (2/5 patients) received neoadjuvant therapy prior to surgery. 60% (3/5 patients) had R_0_ resection (microscopically margin negative) and the rest had unknown margin status. All stage I–II patients received adjuvant therapy. Among these patients, 40% and 60% (2/5 patients, 3/5 patients) had local and distant recurrence, respectively. Of the 4 patients with stage III disease at diagnosis, all received neoadjuvant therapy. One patient went on to undergo R_1_ resection (microscopically margin positive) followed by two cycles of postoperative therapy, followed by recurrent disease. The majority of these patients (3/4 patients, 75%) received second- or third-line immunotherapy as part of a trial and one patient (25%) received immune-based treatment outside of a trial upon disease progression. Five patients with stage IV disease at diagnosis received 1–3 lines of palliative chemotherapy prior to receiving immunotherapy. 40% (2/5 patients) received immunotherapy upon disease progression outside a trial and 60% (3/5 patients) received immunotherapy as part of a trial. In summary, all patients received immunotherapy only after one or more lines of chemotherapy and were progressing with conventional chemotherapy.

The details associated with immunotherapy are provided in Supplementary Table 1. Single agent immunotherapy included pembrolizumab (PD-1 inhibitor; 50%, 7/14 patients), nivolumab (PD-1 inhibitor; 14.5%, 2/14 patients), cabiralizumab (CSF-1R inhibitor; 7%, 1/14 patients), and atezolizumab (PD-1 inhibitor; 7%, 1/14 patients). Combination immunotherapy included ipilimumab (CTLA-4 inhibitor) with nivolumab (14.5%, 2/14 patients), and cabiralizumab with nivolumab (7%, 1/14 patients). The most common adverse effects reported were mild (Grade 1 to 2), and included fatigue (28%, 4/14 patients) and rash (28%, 4/14 patients). Only one patient (7%) had a grade 3 adverse effect (rash) which led to stoppage of immunotherapy. Patients received a median duration of 367 days of immunotherapy (interquartile range: 292–541 days).

### Radiological and biochemical response

**Figure 3:**
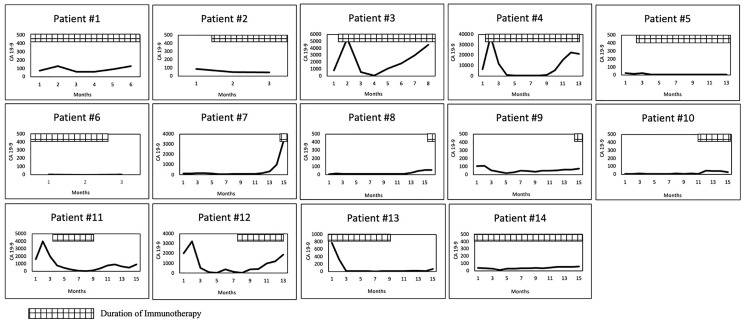
CA 19-9 response following immunotherapy. Y axis represents CA 19-9 levels in U/ml.

**Figure 4:**
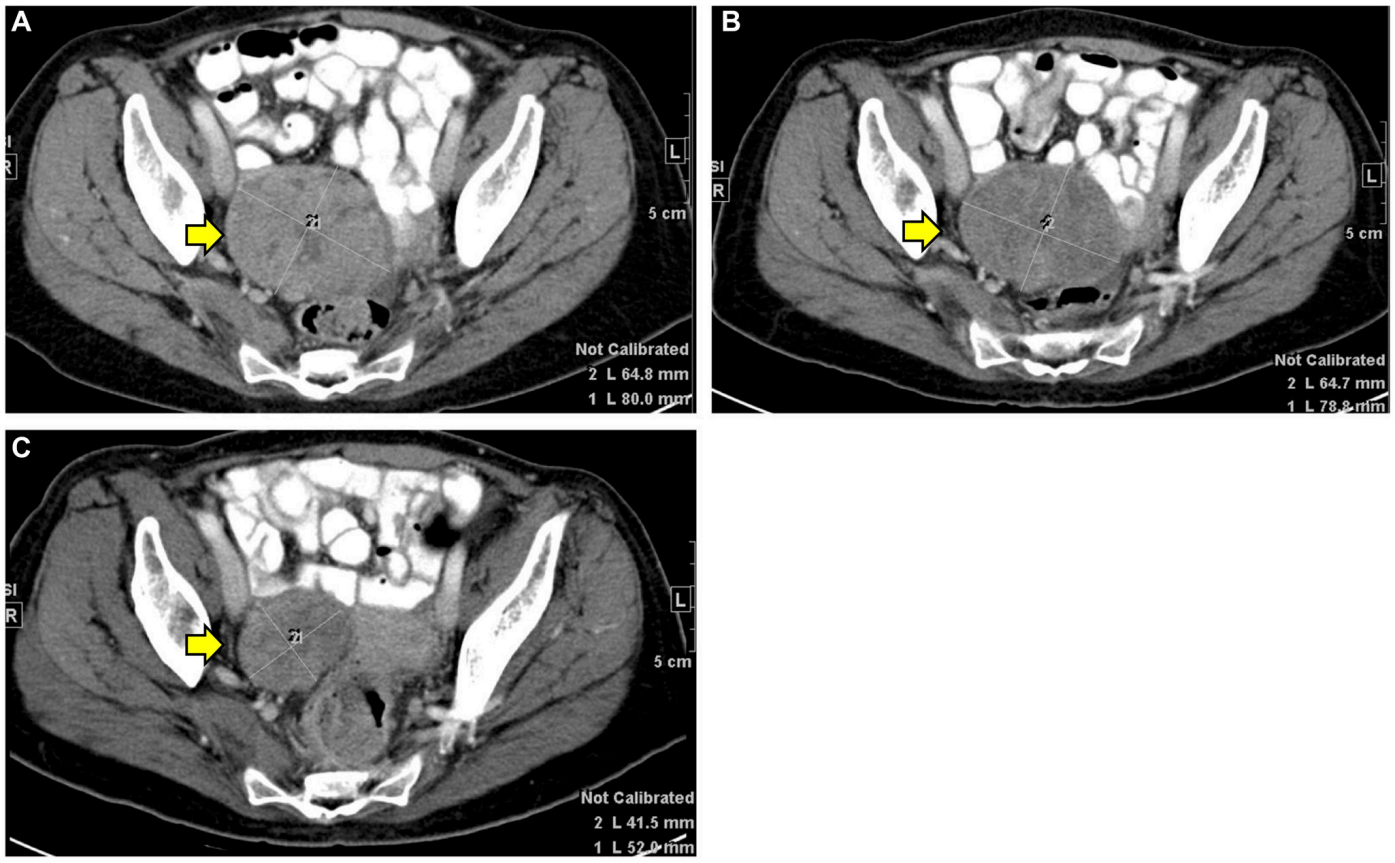
CT imaging findings of patient #9. (**A**) before starting immunotherapy, (**B**) at the start of immunotherapy, and (**C**) at maximal response to immunotherapy.

The radiological response data are described in Supplementary Table 2. Three patients did not have imaging information. None of the patients showed CR, nine patients (82%) showed PR, and two patients (18%) showed PD. Though these two patients had PD (patient #3 and #11), they demonstrated a biochemical response with a fall in CA 19-9 after starting immunotherapy, and hence were included in the analysis (Figure 3). Figure 4 depicts the imaging for a metastatic lesion in the pelvis of patient #9 before, at the start of, and at the maximal response of immunotherapy. The temporal trends in CA 19-9 following immunotherapy are shown in Figure 3. Nearly 67% (8/14 patients) showed stable CA 19-9 levels less than 100 U/ml throughout receipt of immunotherapy. 33% of patients (4/14 patients) had marked reduction in CA 19-9 levels; for instance, patient #3 showed reduction in CA 19-9 from 6418 U/ml to 274 U/ml at maximal response to immunotherapy.

### Survival analysis

**Figure 5:**
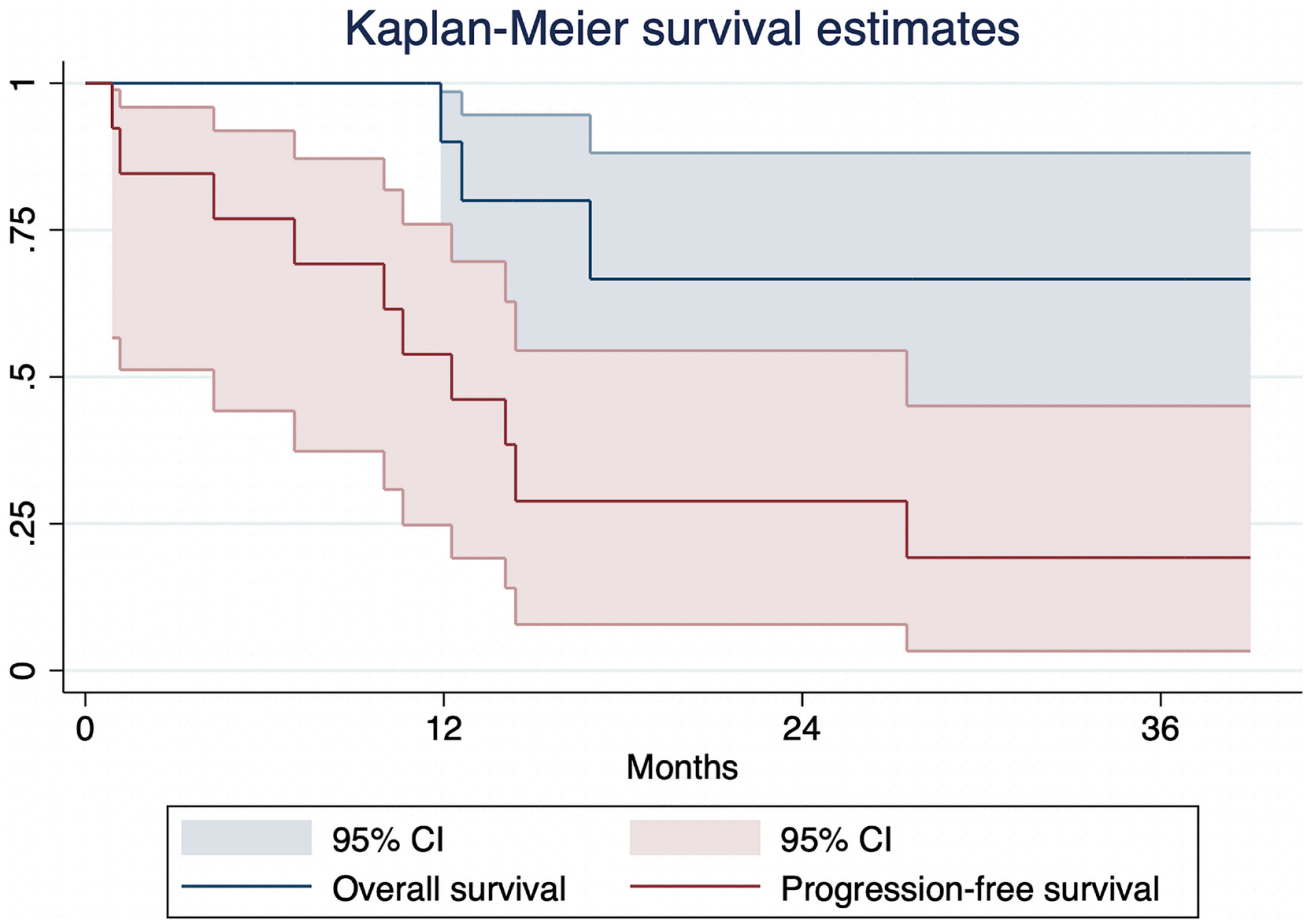
Kaplan Meier curve for overall survival and progression-free survival.

The median follow-up duration was 14.8 months. Kaplan Meier curves for OS and PFS are shown in Figure 5. The median survival for the cohort was not reached. Two of the 14 patients (14%) died, with the remaining still alive at the time of data collection. Almost all patients progressed, with a median PFS of 12 months.

A univariate cox regression analysis was performed comparing patient and treatment factors with survival **(**Supplementary Table 3). None of the factors were associated with OS. Younger age was associated with worse PFS (age 60–69 versus age 50–59 years, hazard ratio (HR): 0.07 (95% confidence interval (CI): 0.006–0.86, *p* = 0.04). Distant disease at initial diagnosis was associated with worse PFS (HR: 6.96, 95% CI: 1.49–32.38, *p* = 0.01).

### Subgroup analysis based on MSI status

Notably, of those with MSI-stable status, patients #4, #8, #13, and #14 were the most remarkable responders with OS ranging between 18 and 36.8 months, and PFS between 14 and 36.8 months. Patients #4 and #13 had a significant drop in CA 19-9 after starting immunotherapy, while rest had stable CA 19-9 levels <200 U/ml. Of these patients, two patients that had imaging data (#8 and #14) had a partial response to immunotherapy.

A subgroup survival analysis was done comparing MSI-high and MSI-stable patients (Supplementary Figure 1). There was no difference in OS (HR: 2.75, 95%CI: 0.24–31.04, *p* = 0.41) and PFS (HR: 0.43, 95% CI: 0.11–1.71, *p* = 0.24) on univariate cox proportional regression analysis.

### Comparative analysis with FOLFIRINOX arm of ACCORD trial

A comparative analysis was performed between patients in this cohort and patients with advanced PDAC in the FOLFIRINOX (5-flurouracil, oxaliplatin, irinotecan, folinic acid) arm of the ACCORD trial [23] using digitalized Kaplan-Meir curves estimated using Webplot digitalizer [24, 25] (Supplementary Figures 2 and 3). There was a significant difference in OS between groups (HR: 2.17, 95% CI: 1.37–3.43, *p* < 0.05). There was no difference in PFS between groups (HR: 0.63, 95% CI: 0.38–1.04, *p* > 0.05).

## DISCUSSION

Multiagent chemotherapy is currently the standard of care for locally advanced and metastatic PC. Neoadjuvant therapy followed by surgical resection offers the best chance for long-term survival for patients with locally advanced disease. However, current evidence shows that even with combination chemotherapy, long-term prognosis is poor. The ACCORD and MPACT trials showed that patients with advanced PDAC who received FOLFIRINOX and gemcitabine/paclitaxel in the first-line, have a median overall survival of 11.1 [26] and 8.5 [27] months, respectively. Once patients reach second-line treatment, the median PFS is around 4 months, and OS is typically an additional 6 months from the start of the second-line treatment regimen [28]. Of the patients with locally advanced disease, only one-third of patients undergo eventual surgical resection, and the median survival in large clinical trials is around 15–18 months after surgery [29–31]. Of the remaining patients who progress, only half of patients are able to receive second-line chemotherapy due to performance status decline. Such inoperable patients have a median OS from the start of second-line therapy of just 2–6 months [32, 33], and median OS from the original date of diagnosis of 4–9 months [32–37]. Thus, novel therapeutic approaches are urgently needed.

Immunotherapy has been investigated as a potential alternative for patients with advanced disease. Though results from numerous phase I–II trials failed to show an apparent benefit, certain isolated cases showing exceptional response to immunotherapy have been reported. Our study adds to this scarce but critical body of literature. Herein, we offer the largest multi-institutional cohort to our knowledge. Though none of the patients showed a complete response, the ORR was 82% (9/11 patients), which is remarkable since the ORR with advanced line chemotherapy is 3–15% [28, 38, 39]. Moreover, 28% (4/14 patients) of patients showed a significant reduction in CA 19-9, despite prior evidence of progression on chemotherapy. The median PFS was 12 months from start of immunotherapy, and the overall survival probabilities at 1 and 2 years were 80% and 70%, respectively. These results are remarkable compared to historical experience with advanced line chemotherapy, where PFS is expected to be under 6 months and OS slightly higher.

Several different immune-oncologic strategies have been tested in PC, including immune checkpoint inhibitors, adoptive T cell transfer therapy, cancer vaccines, and macrophage inhibitors. CTLA-4 inhibits signaling of CD-28, which is a costimulatory protein required for T cell activation and proliferation [40, 41]. Ipilimumab is a CTLA-4 inhibitor previously investigated in patients with PDAC. Royal et al. performed a phase II trial of ipilimumab in advanced PDAC, with no responders identified in a cohort of 27 patients [13]. Similarly, Kamath et al. performed a phase I trial in advanced PDAC receiving second- or third-line therapy. Patients received ipilimumab and gemcitabine, with an ORR of 14% (3/21 patients) and a median OS and PFS of 6.9 and 2.8 months, respectively [42].

The interaction of PD-1 and its ligand PD-L1 in tumor cells inhibits kinase signaling pathways and prevents T-cell activation [43]. Four trials have evaluated and published results using PD-1 inhibitors (pembrolizumab, nivolumab, and durvalimumab), with poor outcomes. Weiss et al. performed a phase II trial of chemotherapy naïve metastatic PDAC patients receiving gemcitabine, paclitaxel, and pembrolizumab with a median PFS and OS of 9.1 and 15 months, respectively [44]. Wainberg et al. performed a phase I study of 50 patients with chemotherapy naïve advanced PDAC receiving nivolumab, paclitaxel, and gemcitabine. The ORR was 18%, median PFS and OS was 5.5 and 9.9 months, respectively [45]. O’Reilly et al. performed a randomized phase II trial comparing durvalumab monotherapy versus durvalumab and tremelimumab (another CTLA-4 antagonist). ORR was just 3.3% (1/34 patients), and 0% (0/32 patients) in the two groups, respectively [15]. The rate of grade 3–4 toxicities ranged between 10.6–76.2% of patients. The outcomes in each of these studies was comparable to the experience with second line chemotherapy alone. In the most encouraging study to date, Le et al. studied the response of 8 patients with MSI-H pancreatic tumors with at least one prior therapy prior to receiving pembrolizumab with a ORR of 53% [43]. Following this, the 2018 ASCO guidelines recommended pembrolizumab for MSI-H pancreatic tumors [46]. Despite these promising findings, it must be acknowledged that MSH-H status in pancreatic cancer is rare, with a prevalence less than 1% [47, 48].

CAR-T cell therapy is a form of adoptive T cell transfer in which harvested patient T cells are re-engineered to target certain tumor genes and are infused back into the patient [49]. A separate technology uses tumor vaccines to induce tumor-specific immunity by administering tumor antigens to patients [50]. Neither CAR-T cell therapy nor tumor vaccines have shown a consistent positive response in patients with advanced PC [51–53]. Tumor-associated macrophages (TAMs) have also been shown to promote an immunosuppressive environment in PC. Nywening et al. conducted a phase I trial investigating FOLFIRINOX with or without the CCL2 inhibitor (PF-04136309) in patients with borderline resectable and locally advanced PDAC. The drug targets the CCL2-CCR2 chemokine axis that recruits TAMs to the tumor resulting in an immunosuppressive environment and immune escape. The trial has been one of the more favorable for conventional PDAC, with an ORR observed in 49% of patients (16/33) [54], but was an early-phase clinical trial and attribution to immunotherapy cannot be ascertained since patients received FOLFIRNOX (which is associated with a 30–40% response rate) [26].

PC is considered a ‘cold tumor’ compared to the immune response to other cancer types, and the lack of response to immunotherapy to date is primarily attributed to a highly desmoplastic and immunosuppressive environment. The TME (or stromal compartment) is uniquely abundant, comprising 80% of the tumor mass in PC and imparts resistance to therapy by various mechanisms. Hypothesized explanations of the poor immune response include reduced T cell migration related to a dense fibrotic stroma, downregulation of major histocompatibility complex class I (MHC I) molecules, increased signaling of regulatory T cells, reduced cytotoxic T cells, and increased CTLA-4 and PD-1 signaling to downregulate activation of T cells [55]. Also, PDAC tumor cells escape immune surveillance by binding tumor-associated antigens, which are rich in extracellular vesicles and competitively bind autoantibodies, protecting tumor cells from antibody-mediated immunity [56]. One possible method to improve responsiveness of immunotherapy could be to modify the tumor microenvironment by reducing desmoplasia and restoring immune surveillance, although this strategy seemed to have an unintended opposite response in mice where PDAC aggressiveness increased when the stroma was targeted [55, 57]. The combination of immune checkpoint inhibitors and stroma-targeting agents like clodronate liposomes which inhibit tumor associated macrophages may improve overall responsiveness [58]. Additionally, low tumor mutational burden has also been proposed as a driving factor of immune tolerance in PC.

Recently, case reports have shown examples of exceptional responses to immunotherapy. Patil et al. described a patient with Lynch syndrome and MSI-high PDAC metastatic to the liver, who received pembrolizumab following disease progression after two lines of chemotherapy. The patient had a complete clinical and pathological response that lasted up to 11 months after a single cycle of pembrolizumab [20]. Ye et al. studied the treatment response in a patient with MSI-stable metastatic PDAC with SMAD2 and TSC2 gene mutations following two lines of chemotherapy. This patient received a combination of S-1 chemotherapy (tegafur, gimeracil, oteracil) and a PD-1 inhibitor (sintilimab), and a partial response to therapy was observed that continued for 8 months following initial therapy. TSC2 is a tumor suppressor gene and impaired expression has been shown previously to be associated with improved responsiveness to checkpoint inhibitors, although the reason for this response is still unknown [59]. The TGF-beta/SMAD 4 signaling pathway is believed to be important to the tumor immune response, since TGF-beta can enhance expression of PD-1 and suppress the immune response [60]. Lundy et al. studied the response of a patient with metastatic PDAC and a BRCA2 mutation, along with high tumor mutational burden to gemcitabine, paclitaxel, pembrolizumab, and olaparib (a PARP inhibitor). This patient showed a complete clinical response to treatment [19]. Of note, nearly 5–9% of all patients with PDAC have BRCA 1 and BRCA 2 mutations [7].

Our case series showcases the usefulness of immunotherapy in a subset of patients with advanced PDAC. On comparative analysis, this cohort of exceptional responders had a better survival compared to the FOLFIRINOX arm of the ACCORD trial. Though it may not be possible to make direct comparisons and conclusions from this rudimentary analysis due to differences in patient population and line of therapy, it provides a summary of the magnitude of difference in outcomes. Several features of these tumors and patients in this cohort are worth emphasizing. Only 43% of patients had MSI-H status. Thus, while MSI-H is likely the most important predictor of response (1% of all PDAC patients have MSI-H), the majority of responders were actually MSI-stable. While mutational burden is a known predictor of PD-1 inhibition in other cancers (e.g., lung) [61, 62], the proportion of patients with a smoking history in this cohort was very low. Two of the patients had a history of ovarian or breast cancer, pointing to a possible BRCA mutation in the PC, and indicating a genetic susceptibility to chromosome instability and immune-oncologic responsiveness, although BRCA status was not obtained in this cohort.

Several possible factors may be responsible for the exceptional response to immunotherapy in patients with MSI-stable status which are summarized in Supplementary Figure 4. Tumor-intrinsic oncogenic signaling like the inactivation of PIK3 pathway through phosphate and and tensin homolog (PTEN) activation has been shown to be associated with better survival in melanoma patients undergoing immunotherapy [63]. Presence of interferon gamma in the tumor has been shown to accentuate immune checkpoint inhibitors [64]. Other possible mechanisms including epigenetic alterations [65], tumor suppressive immune cells like TAMs [66], and immunosuppressive cytokines like transforming growth factor-β have also been implicated [67]. Finally, favorable gut microbiome with certain bacterial species have been associated with a response to immunotherapy in melanoma [68]. On cox proportional regression analysis, younger age and distant disease was associated with worse PFS. Wang et al. performed a retrospective study to identify prognostic factors for patients with metastatic gastrointestinal cancers undergoing treatment with immunotherapy. Younger patients had worse survival [69]. This has been shown in other meta-analyses exploring immune checkpoint inhibitors [70]. The possible reasons are unknown, however few possible reasons could be due to stronger MHC based driver selection [71] and the lower ratio of T cells to T regulatory cells in younger individuals [72]. Further prospective research is necessary to study these mechanisms in PC. Based on the results, the selection criteria for immunotherapy trials investigating pancreatic ductal adenocarcinoma (PDAC) may require revision to encompass a broader range of factors beyond MSI status.

Numerous predictive biomarkers have been associated with a better response to immune checkpoint inhibitors in other solid tumors including tumor mutational burden, PDL-1 protein expression [73–76], density of tumor infiltrating lymphocytes (TILs) [77], HLA class I diversity [78, 79], loss of heterogeneity at HLA class I alleles [80], T cell repertoire clonality change, T cell inflamed microenvironment, tumor-specific mutations, gut microbiome diversity, specific gut microbial species [81–83], TGF-beta expression [84], and mutations in beta-catenin pathway [85]. In addition, systemic markers including neutrophil to lymphocyte ratio, T cell clonality, circulating Treg count, and lactate have been shown to be associated with PFS and OS in several cancer types upon treatment with immunotherapy [86–90]. A possible way to improve patient selection could be to develop a composite predictive model considering these various elements, although the sample size and molecular profiling requirements would be significant. Finally, studies incorporating multi-omics data can provide a greater understanding of prognostic phenotypes and can assist translational research by integrative cancer models [91].

There are several notable limitations to this study. This is a retrospective collection of patients and is subject to recall bias. It may not be representative of the general population and have significant selection bias. Several tumor characteristics including tumor microenvironment characteristics, BRCA mutational status to name a few were unknown. Next generation sequencing may help in identifying molecular basis for response in patients with MSI-stable status. However, this was not available for the cohort. The denominator of PC patients who received immunotherapy was not available. Hence it was not possible to identify factors for an exceptional response to immunotherapy. The subgroup survival analysis based on MSI status is limited by low sample size and considerable large confidence intervals, as indicated by our KM curve analysis. Similarly, the comparative analysis with historical cohort is limited by this and errors of estimating HR from Kaplan Meier curves.

Immunotherapy has failed to lead to a paradigm change in treatment for pancreatic cancer. A transference of knowledge of immunotherapy from other solid tumors to pancreatic cancer has not yielded meaningful and actionable information. Our case series of exceptional responders to immunotherapy emphasizes that a favorable response is possible, but with an unknown biologic explanation in roughly half of the patients (the MSI-stable patients). Further understanding of the tumor microenvironment, immune resistance, and molecular predictors of response are needed to achieve better outcomes with this therapeutic approach.

## MATERIALS AND METHODS

### Patient selection

This multi-institutional case series was reviewed and approved by the University Hospitals, Cleveland Medical Center Institutional review board (IRB no. 20191439). Patients diagnosed with pancreatic ductal adenocarcinoma and histological variants who had an exceptional response to immunotherapy were included. Exceptional responders were defined as those having either a partial (PR) or complete response (CR) based on the Immune-Modified Response Evaluation Criteria in Solid Tumors (imRECIST) imaging criteria [92] or a biochemical response (decreasing or stable trend of CA 19-9 levels) following start of immunotherapy. Immunotherapy regimens included immune checkpoint inhibitors, tumor vaccines, CAR T-cell therapy, and macrophage receptor blockers. Patients who received chemotherapy along with immunotherapy were excluded to isolate any therapeutic benefit to the immune-oncologic treatment.

### Data collection

Oncologists from major cancer centers in the United States were contacted to submit data from patients with PC with the previously mentioned inclusion criteria. Among the institutions that responded, a data use agreement was signed between institutions to share deidentified patient information. Data were shared and stored through the Research Electronic Data Capture (REDCap) web application [93].

The following information was recorded for all patients: age, gender, race, history of cancer, history of diabetes, family history of cancer, smoking history, Eastern Cooperative Oncology Group (ECOG) functional status, histological diagnosis, genetic mutation, mutational burden status, stage of disease at diagnosis, and start of immunotherapy, resection status, and the use of other prior treatments (chemotherapy or radiation). Pathological information among those who underwent resection included margin status, lymph node status, grade of tumor, and venous, lymphatic, or perineural invasion. All patients were diagnosed with invasive adenocarcinoma of the pancreas, although specific diagnoses may include variants of conventional pancreatic ductal adenocarcinoma (PDAC) [94]. Precise histologic diagnoses and subtypes are indicated in the results. Details around immunotherapy were recorded, included the treatment type and adverse effects according to the Common Terminology Criteria for Adverse Events (CTCAE) as grade 1 to 4 toxicity [95].

### Measured outcomes

The outcomes of the study included overall survival (OS), progression free survival (PFS), CA19-9 response, and radiological response. OS was calculated from the start date of immunotherapy until the last follow up date or death. PFS was calculated from the start date of immunotherapy to the date of progression or death. Kaplan Meier curves were used to graphically depict OS and PFS. CA 19-9 values prior to, during, and after immunotherapy were collected and temporally plotted to assess response to therapy. A univariate cox regression analysis was performed to assess the association between patient and treatment factors with OS and PFS respectively. Radiological response to immunotherapy was classified as CR, PR, stable disease (SD), or progressive disease (PD), according to standard definitions [96]. All statistical analyses were performed using StataSE v16.0 (Statacorp LLC, College Station, TX, USA) and a *p*-value less than 0.05 was used to indicate statistical significance.

## SUPPLEMENTARY MATERIALS


