# Supplementary material for: Exceptional responders to immunotherapy in pancreatic cancer: A multi-institutional case series of a rare occurrence
Source: Oncotarget. 2025 Jun 10;16:427–42. doi: 10.18632/oncotarget.28739 (PMC12151408; doi:10.18632/oncotarget.28739)
Supplement: Supplementary file 1 [file oncotarget-16-28739-s001.pdf]

Exceptional responders to immunotherapy in pancreatic cancer: A multi-institutional case series of a rare occurrence

SUPPLEMENTARY MATERIALS

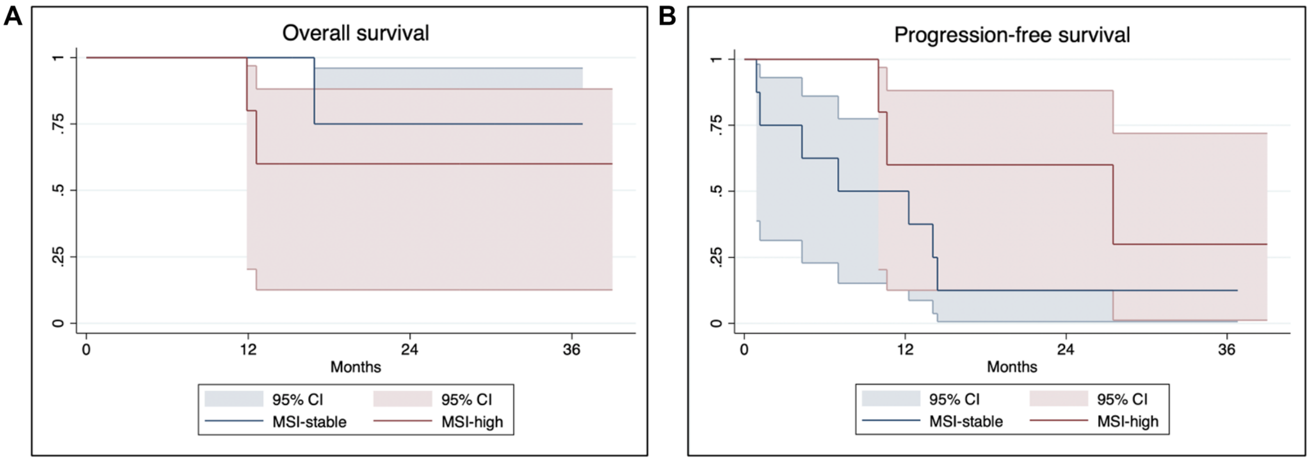

Supplementary Figure 1: Subgroup survival analysis based on microsatellite instability. Cox regression analysis: (**A**) Overall survival: Hazard ratio (HR): 2.75, 95% confidence interval (CI): 0.24–31.04, $p = 0.41$); (**B**) progression free survival: HR: 0.43, 95% CI: 0.11–1.71, $p = 0.24$).

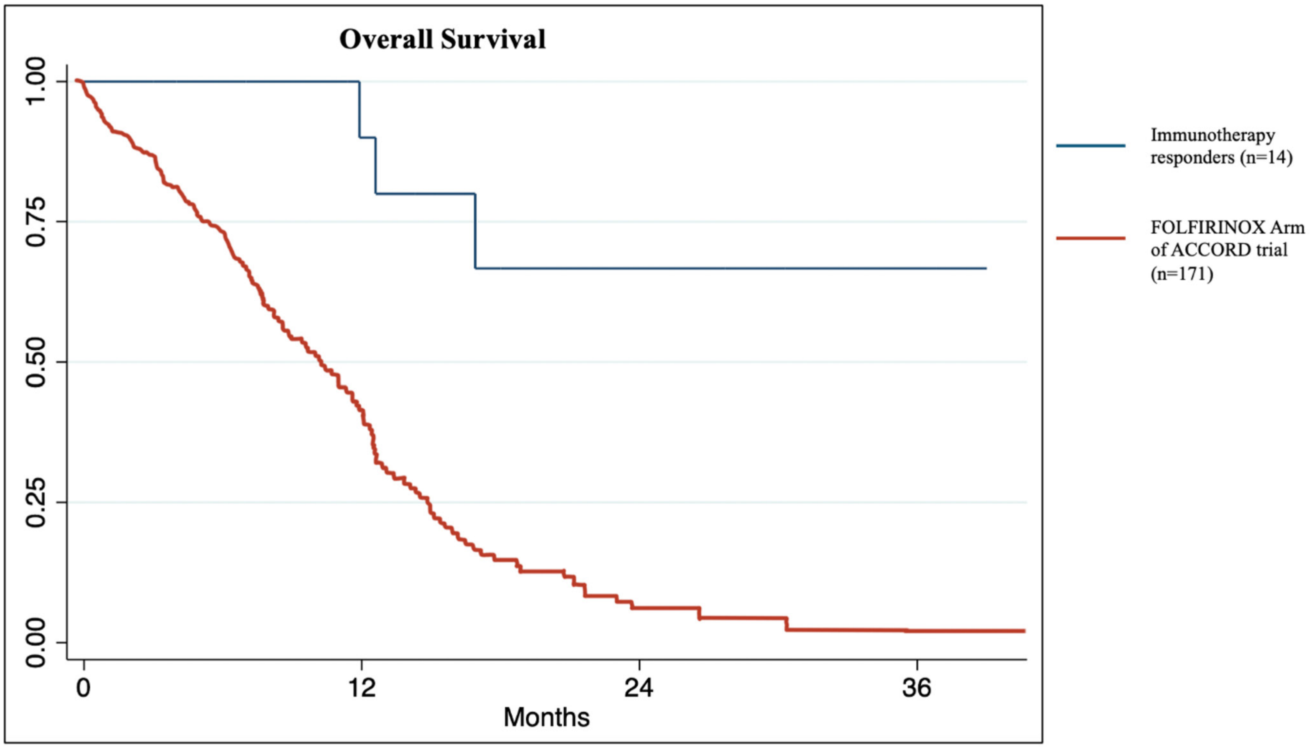

Supplementary Figure 2: Overall survival compared with FOLFIRINOX arm of ACCORD trial. Cox regression analysis: Overall survival: Hazard ratio (HR): 2.17, 95% confidence interval: 1.37–3.43, $p < 0.05$).

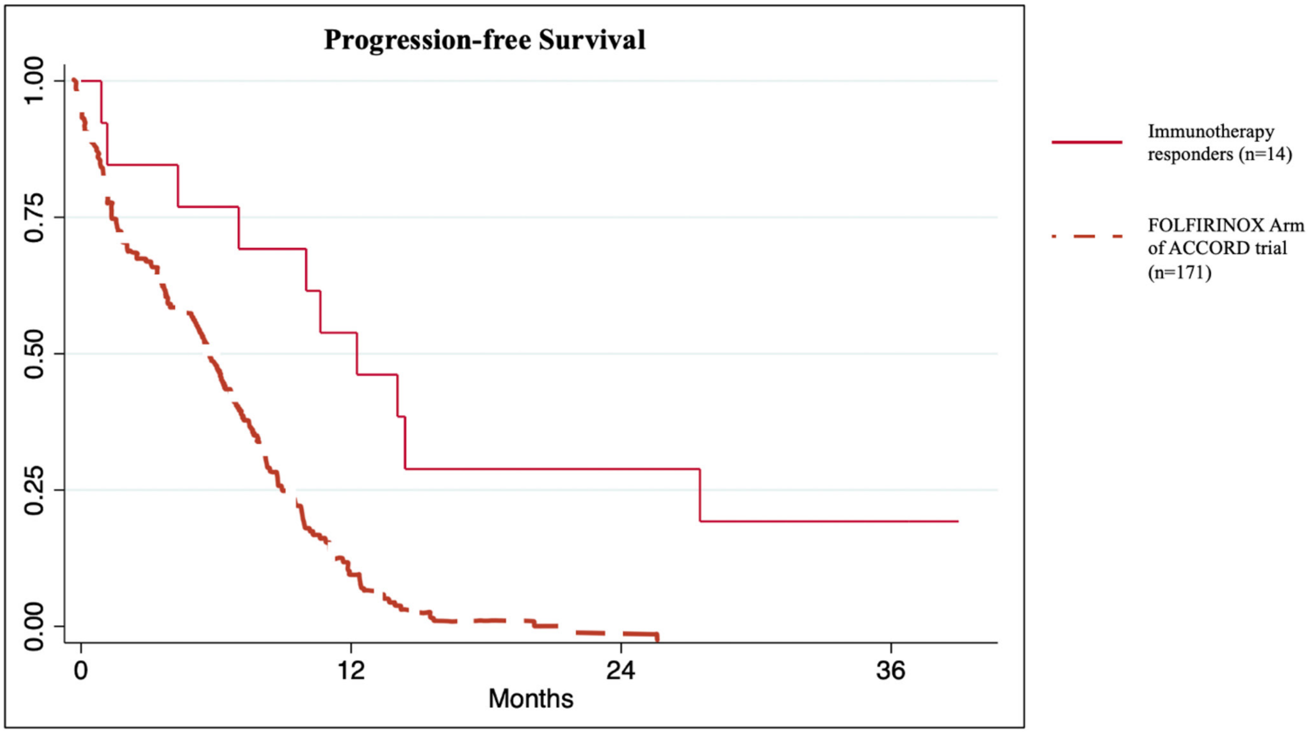

Supplementary Figure 3: Progression-free survival compared with FOLFIRINOX arm of ACCORD trial. Cox regression analysis: Progression-free survival: Hazard ratio (HR): 0.63, 95% confidence interval: 0.38–1.04, $p > 0.05$).

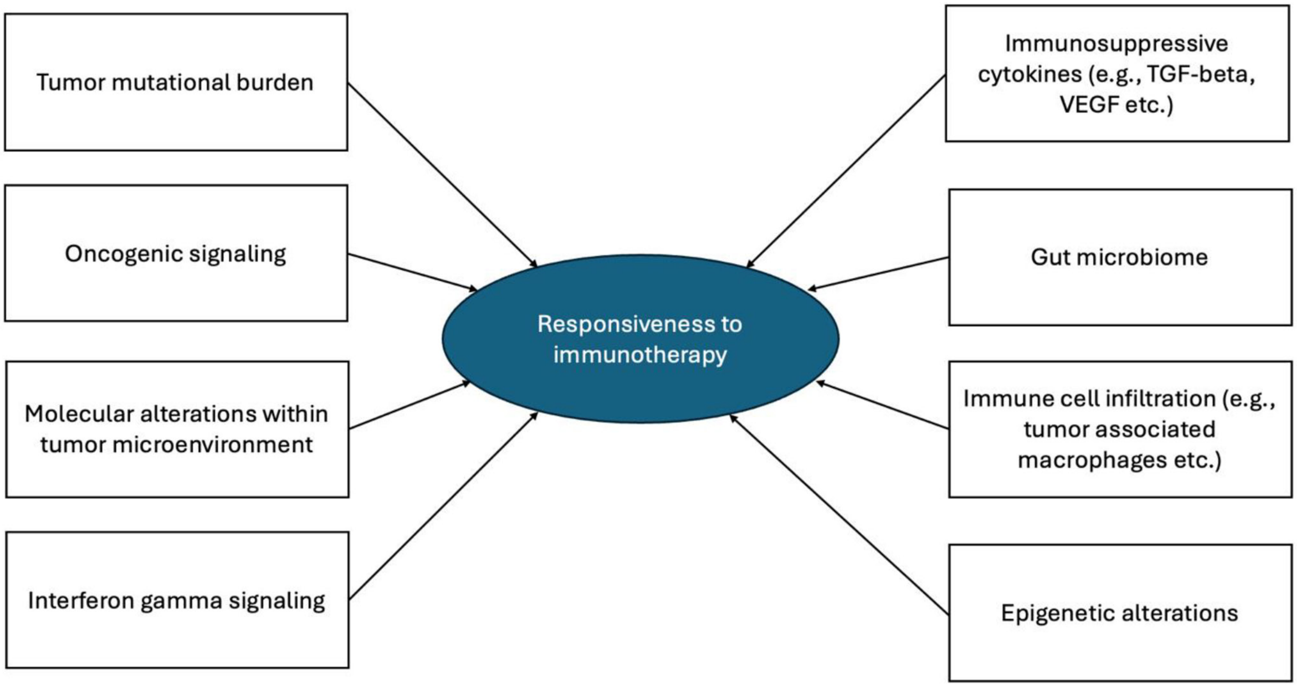

Supplementary Figure 4: Summary of possible mechanisms of exceptional response to immunotherapy.

Supplementary Table 1: Immunotherapy treatment characteristics

Parameter	*n* (%) or Median (IQR)
**Single agent immunotherapy**	
Pembrolizumab (PD-1 inhibitor)	7/14 (50%)
Nivolumab (PD-1 inhibitor)	1/14 (7%)
Cabiralizumab (CSF-1R inhibitor)	1/14 (7%)
Atezolizumab (PD-1 inhibitor)	1/14 (7%)
**Combination immunotherapy**	
Ipilimumab (CTLA4 inhibitor) + Nivolumab (PD-1 inhibitor)	2/14 (14.5%)
Cabiralizumab + Nivolumab	2/14 (14.5%)
**Duration of therapy (days)**	367 (292–541)
**Indication of starting immunotherapy**	
Disease progression/recurrence	5/14 (36%)
Enrolled in a trial	9/14 (64%)
**Adverse effects reported***	
Fatigue (Grade 1–2)	4/14 (28%)
Rash (Grade 1–3)	4/14 (28%)
Photosensitivity (Grade 1)	1/14 (7%)
Lower extremity edema (Grade 1)	1/14 (7%)
Back pain (Grade 1)	1/14 (7%)
Peripheral neuropathy (Grade 1)	1/14 (7%)
**Reason for stopping immunotherapy**	
Disease progression	9/14 (64%)
Adverse effects	1/14 (7%)
Unknown	4/14 (28%)

*Adverse effects reported according to Common Terminology Criteria for Adverse Events (CTCAE). Abbreviations: PD-1: programmed cell death 1; CTLA4: cytotoxic T lymphocyte-associated protein 4, CSF-1R: Colony Stimulating Factor – Receptor 1.

Supplementary Table 2: Radiological response following immunotherapy

Patient identifier*	Diameter of pancreatic lesion before start of immunotherapy (mm)	Sum of diameter of metastatic lesion/s before start of treatment (>10 mm)	Sum of short axis of lymph nodes before start of immunotherapy (>15 mm)	Diameter of pancreatic lesion at maximal response of immunotherapy (mm)	Sum of diameter of metastatic lesion/s at maximum response following immunotherapy (>10 mm)	Sum of short axis of lymph nodes at time point of maximum response (>15 mm)	imRECIST (% decrease in sum of diameters of target lesion/s)
2	35	0	0	11	0	0	PR (−69%)
3	0	14 + 28 + 24	13	0	21 + 34 + 30	13	PD (+24%)
5	37	0	0	25	0	0	PR (−32%)
6	80	0	0	12	0	0	PR (−85%)
7	30	0	39 + 38	16	0	11 + 9	PR (−58%)
8	12	29 + 16	0	7	16 + 12	0	PR (−39%)
9	45	80	0	21	52	0	PR (−42%)
10	55	11	0	38	0	0	PR (−43%)
11	150	330	130	280	450	110	PD (+38%)
12	300	50	0	150	50	0	PR (−33%)
14	78	41	0	51	31	0	PR (−31%)

*Imaging information was unavailable for patients 1, 4, and 13. Abbreviations: PR: partial response; SD: stable disease; PD: disease progression; imRECIST: Immune-Modified Response Evaluation Criteria in Solid Tumors.

Supplementary Table 3: Patient factors associated with overall and progression-free survival

Parameter	Overall survival		Progression-free survival	
	**HR (95% CI)**	***p*-value**	**HR (95% CI)**	***p*-value**
Age				
50–59 years	Reference		Reference	
60–69 years	0.72 (0.06–8.25)	0.79	**0.07 (0.006–0.86)**	0.04
>70 years	1	–	0.08 (0.006–1.11)	0.06
Male gender	2.15 (0.19–23.87)	0.53	1.71 (0.46–6.34)	0.42
Microsatellite instability-high	2.75 (0.24–31.04)	0.41	0.43 (0.11–1.72)	0.23
Diabetes Mellitus	0.61 (0.05–6.81)	0.69	0.59 (0.15–2.40)	0.47
History of previous cancer	3.74 (0.23–59.91)	0.35	0.63 (0.07–5.11)	0.66
Family history of cancer	0.34 (0.03–3.85)	0.38	1.06 (0.29–3.89)	0.91
History of smoking	3.74 (0.23–59.12)	0.35	0.85 (0.10–6.96)	0.88
Stage at diagnosis	1.62 (0.47–5.59)	0.44	1.51 (0.66–3.45)	0.32
Distant versus localized disease at diagnosis	8.27 (0.74–91.75)	0.09	**6.96 (1.49–32.38)**	**0.01**
ECOG functional status	1	–	0.33 (0.09–1.16)	0.08
Surgery	0.31 (0.03–3.56)	0.35	0.71 (0.20–2.50)	0.59
Margin status	1	–	2.44 (0.15–39.72)	0.53
Grade	0.52 (0.06–4.03)	0.54	1.53 (0.68–3.66)	0.28
Venous/lymphatic invasion	1	–	0.98 (0.16–6.12)	0.99
Perineural invasion	1	–	0.35 (0.04–3.16)	0.35
Stage at start of immunotherapy	1	–	6.21 (0.76–51.51)	0.08
Combination immunotherapy	0.77 (0.06–8.59)	0.83	0.45 (0.09–2.15)	0.32

Abbreviations: ECOG: Eastern Cooperative Oncology Group; HR: hazard ratio; CI: confidence interval.
